# Life's Essential 8 and Parkinson's Disease Risk: A Cross-Sectional Study Based on NHANES Data (2005–2018)

**DOI:** 10.31083/RN38937

**Published:** 2025-12-24

**Authors:** Jing Liu, Bo Gao, Li-Jun Ma, Xi-bin Gao

**Affiliations:** ^1^Department of Neurology, The Affiliated Hospital of Yan'an University, 716000 Yan'an, Shaanxi, China; ^2^Department of Laboratory Medicine, The Affiliated Hospital of Yan'an University, 716000 Yan'an, Shaanxi, China

**Keywords:** Parkinson's disease, cardiovascular health, health behavior, cross-sectional studies, propensity score, enfermedad de Parkinson, alud cardiovascular, comportamiento saludable, estudios transversales, puntuación de propensión

## Abstract

**Objective:**

Existing research on the link between Life's Essential 8 (LE8) and the risk of Parkinson's disease (PD) remains limited. This study aimed to elucidate how LE8 relates to PD risk among USA adults aged 40 and above.

**Methods:**

Data were derived from the 2005–2018 National Health and Nutrition Examination Survey (NHANES). Propensity score matching (PSM) was employed to control for selection bias. Multivariable logistic regression was applied to assess the association between LE8 and PD prevalence, while restricted cubic spline (RCS) modeling was adopted to explore potential relationships. Additionally, subgroup analyses were conducted to further examine the connection between LE8 and PD.

**Results:**

A total of 18,270 participants were included, among whom 259 reported having PD. An inverse association was observed between LE8 and PD. Prior to matching, the odds ratio (OR) for per 1 point increase in LE8 was 0.98, and 0.97 after matching. Compared with individuals with low LE8 scores (<50), those with moderate scores (50–79) had a PD OR of 0.62 before matching and 0.52 after matching. Participants with high LE8 scores (≥80) observed a PD OR of 0.43 prior to matching and 0.32 post-matching. RCS curves suggested a non-linear inverse trend. Subgroup analyses revealed a consistent inverse association between LE8 scores and PD risk across the majority of strata.

**Conclusion:**

Among adults aged 40 and older, LE8 was inversely correlated with PD prevalence. Given the cross-sectional design, causal relationships cannot be inferred; however, the findings suggest that lifestyle modifications may aid in PD prevention and warrant further investigation in prospective studies.

## Abstract (es)

**Objetivo:**

Las investigaciones existentes sobre la relación entre los 8 elementos esenciales para la vida (LE8, *Life’s Essential 8*) y el riesgo de padecer la enfermedad de Parkinson (EP) siguen siendo limitadas. El objetivo de este estudio es esclarecer cómo se relacionan los LE8 con el riesgo de padecer EP entre los adultos estadounidenses de 40 años o más.

**Métodos:**

Los datos se obtuvieron de la Encuesta Nacional de Examen de Salud y Nutrición (NHANES, *National Health and Nutrition Examination Survey*) de 2005–2018. Se utilizó el emparejamiento por puntuación de propensión (PSM, *Propensity Score Matching*) para controlar el sesgo de selección. Se aplicó una regresión logística multivariable para evaluar la asociación entre los LE8 y la prevalencia de la EP, mientras que se adoptó un modelo de spline cúbico restringido (RCS, *restricted cubic spline*) para explorar las posibles relaciones. Además, se realizaron análisis de subgrupos para examinar más a fondo la conexión entre los LE8 y la EP.

**Resultados:**

Se incluyó a un total de 18.270 participantes, de los cuales 259 informaron tener EP. Se observó una asociación inversa entre los LE8 y la EP. Antes del emparejamiento, la oportunidad relativa (OR, *odds ratio*) por cada aumento de 1 punto en los LE8 era de 0,98, y de 0,97 después del emparejamiento. En comparación con las personas con puntuaciones bajas en los LE8 (<50), aquellas con puntuaciones moderadas (50–79) tenían una OR de EP de 0,62 antes del emparejamiento y de 0,52 después del emparejamiento. Los participantes con puntuaciones altas en LE8 (≥80) observaron una OR de EP de 0,43 antes del emparejamiento y de 0,32 después del emparejamiento. Las curvas de RCS sugirieron una tendencia inversa no lineal. Los análisis de subgrupos revelaron una asociación inversa constante que está presente en la mayoría de los estratos entre las puntuaciones de LE8 y el riesgo de EP.

**Conclusión:**

Entre los adultos de 40 años o más, el LE8 se correlacionó inversamente con la prevalencia de la EP. Dado el diseño transversal, no se pueden inferir relaciones causales, pero los hallazgos sugieren que las modificaciones en el estilo de vida pueden ayudar a prevenir la EP y justifican una investigación más profunda en estudios prospectivos.

## 1. Introduction

As a prevalent neurodegenerative disorder, Parkinson’s disease (PD) is a neurodegenerative disorder marked by the selective deterioration of dopaminergic neuronal pathways. This neuronal loss causes severe disability and poses significant public health challenges worldwide due to its motor, non-motor, and cognitive symptoms [1, 2, 3]. PD, as the second most prevalent neurodegenerative disorder worldwide, is second to Alzheimer’s disease. Over the last 20 years, its incidence and prevalence have risen rapidly, making PD the fastest-growing neurological condition globally [4]. Research shows that the primary cause of PD involves an intricate interplay of genetic and environmental factors [5, 6]. Unlike genetic factors, which remain stable, environmental factors are subject to change over time. For example, lifestyle changes in recent decades have contributed to a rise in obesity, metabolic syndrome, insulin resistance, and chronic inflammation, all of which are closely linked to the development and progression of PD [7]. Moreover, Western dietary patterns and alterations in gut microbiota may drive PD-related neurodegeneration via the gut-brain axis, involving the transport of α-synuclein [8]. Thus, a thorough analysis of how lifestyle factors relate to PD is critical.

In 2010, the American Heart Association introduced a framework known as “Life’s Simple 7” (LS7), comprising seven key metrics—three behavioral factors and four physiological indicators—designed to evaluate and promote cardiovascular health (CVH) at both individual and population levels. The components of LS7 include dietary habits, tobacco use, physical activity, body mass index (BMI), arterial blood pressure, serum cholesterol, and fasting plasma glucose [9]. Over time, the AHA recognized sleep health as a crucial component, leading to the development of a novel algorithm, scoring from 0 to 100, to quantify each metric, now termed “Life’s Essential 8” (LE8). This approach comprehensively takes into account health behaviors, metabolic factors, and individual differences [10]. Evidence from previous studies indicates that maintaining optimal LS7 scores may reduce the risk of various chronic illnesses [11, 12, 13]. Emerging research suggests that LE8 can more effectively correlate with the onset and progression of numerous diseases [14, 15, 16, 17, 18].

Although LE8 has demonstrated significant associations and potential in research on heart disease, stroke, and various chronic conditions, its connection with PD remains poorly understood. Given the scarcity of systematic studies on the connection between LE8 and PD, the present work seeks to address this gap using cross-sectional data.

## 2. Methods and Materials

### 2.1 Study Population

Conducted in the USA, National Health and Nutrition Examination Survey (NHANES) is a nationally representative, ongoing cross-sectional survey aimed at monitoring health and nutritional status. Utilizing a stratified, multistage probability design, NHANES collects nationally representative health data on a biennial basis. Initial data are collected through structured, in-person interviews at participants’ homes, followed by clinical assessments at Mobile Examination Centers, where biological specimens such as blood and urine are collected. The study protocol was reviewed and approved by the NCHS Research Ethics Review Board, and all individuals provided informed consent before participation.

**Fig. 1.**
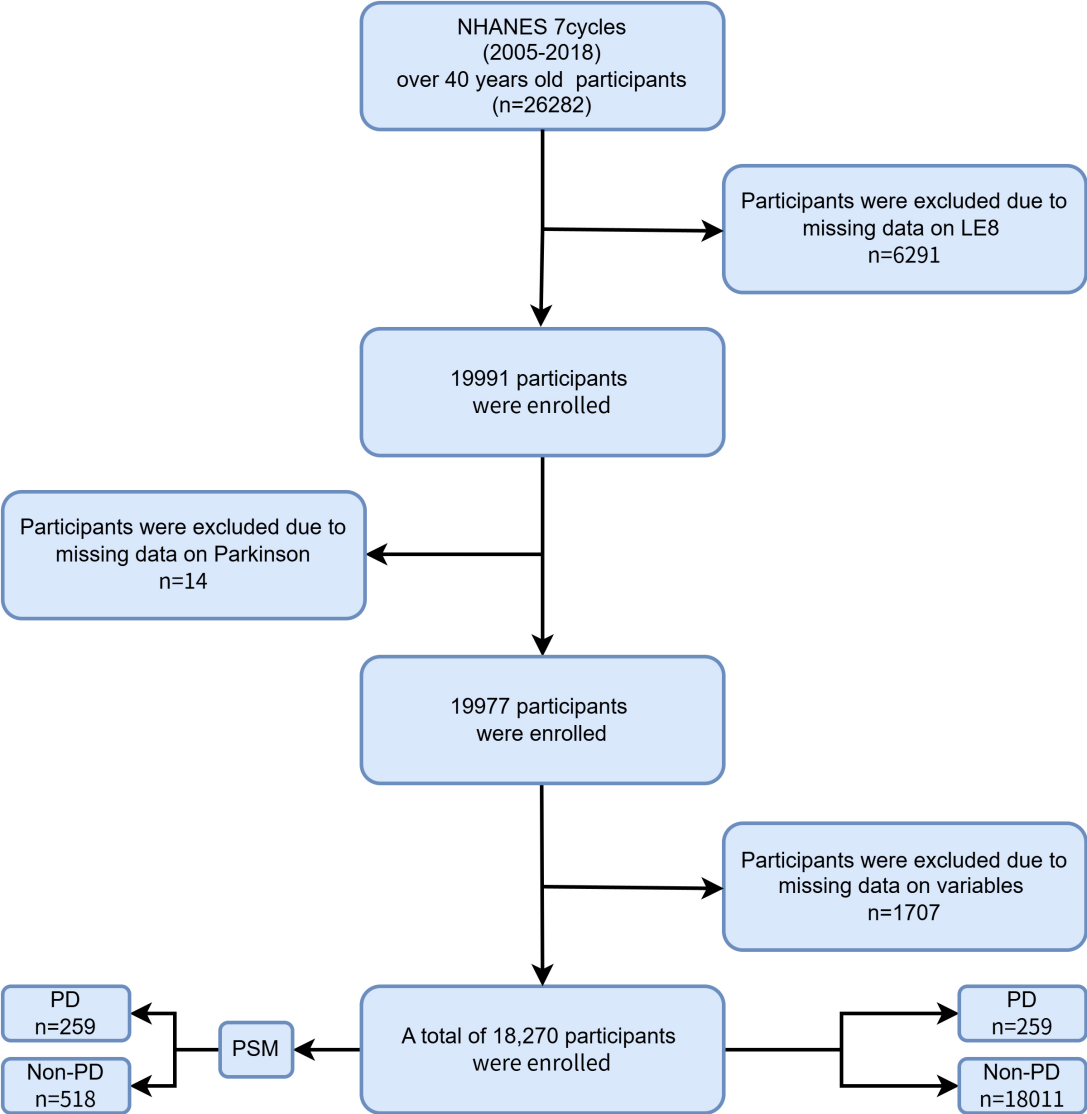
**Flow chart of participant selection from NHANES 2005–2018**. NHANES, National Health and Nutrition Examination Survey; LE8, Life’s Essential 8; PD, Parkinson’s disease; PSM, propensity score matching.

For this analysis, data were obtained from seven NHANES survey cycles spanning 2005 to 2018. The initial sample comprised 26,282 individuals aged 40 years or older. Exclusion criteria included missing Life’s Essential 8 (LE8) metrics (n = 6291), absence of information regarding PD medication use (n = 14), and incomplete data on essential covariates (n = 1707). After applying these criteria, a total of 18,270 participants remained eligible for inclusion. Fig. 1 provides a detailed schematic of the participant selection process.

### 2.2 Assessment of PD

In the NHANES database, participants with PD were identified based on their use of “anti-Parkinson’s medications” [19, 20]. This identification method relied on responses to prescription medication questions. Participants receiving treatment for PD were identified as PD cases, whereas those not receiving such treatment were categorized as non-PD cases.

### 2.3 Assessment of LE8

The LE8 construct comprises eight components, including four health behaviors: sleep duration, tobacco exposure, physical activity, and dietary quality as well as four biological metrics: systolic and diastolic blood pressure, fasting glucose levels, non-high-density lipoprotein (non-HDL) cholesterol, and BMI.

Participant information was primarily collected through self-report and included variables such as sleep duration, secondhand smoke exposure, active tobacco use, weekly physical activity levels, medication use, dietary habits, and diabetes status. Dietary quality was evaluated using the Healthy Eating Index–2015 (HEI–2015). In addition, anthropometric and laboratory measurements-including height, weight, blood pressure, and fasting glucose-were obtained in accordance with NHANES standardized procedures.

BMI was calculated as weight (kg) divided by the square of their height (m^2^). Blood pressure readings-including both systolic and diastolic values-were obtained by calculating the mean of three consecutive measurements. Levels of non-HDL cholesterol were estimated by subtracting HDL cholesterol from the total cholesterol concentration. Venous blood specimens were collected and transported to certified laboratories for biochemical analysis, including assessments of fasting plasma glucose, lipid panels, and glycated hemoglobin (HbA1c).

The scoring methodology for LE8, applied to NHANES data, is summarized in Table 1. Each of the eight components was assigned a value ranging from 0 to 100, and the overall LE8 score was computed as the arithmetic mean of these individual scores. A higher total score reflected more favorable CVH. For analytical purposes, LE8 scores were stratified into three categories: poor CVH (0–49), intermediate CVH (50–79), and ideal CVH (80–100) [21].

**Table 1.** **Definition and scoring approach for the American Heart Association’s LE8**.

Domain	CVH Metric	Measurement	Quantification and Scoring of CVH Metric	
Health Behaviors	Diet	Healthy Eating Index-2015 diet score percentile	Quantiles of DASH-style diet adherence	
			Scoring (Population):	
			Points	Quantile
			100	≥95th percentile (top/ideal diet)
			80	75th–94th percentile
			50	50th–74th percentile
			25	25th–49th percentile
			0	1st–24th percentile (bottom/least ideal quartile)
	Physical activity	Self-reported minutes of moderate or vigorous physical activity per week	Metric: Minutes of moderate (or greater) intensity activity per week	
			Scoring:	
			Points	Minutes
			100	≥150
			90	120–149
			80	90–119
			60	60–89
			40	30–59
			20	1–29
			0	0
	Nicotine exposure	Self-reported use of cigarettes or inhaled nicotine-delivery system	Metric: Combustible tobacco uses and/or inhaled NDS use; or secondhand smoke exposure	
			Scoring:	
			Points	Status
			100	Never smoker
			75	Former smoker, quit ≥5 yrs.
			50	Former smoker, quit 1–<5 yrs.
			25	Former smoker, quit <1 year, or currently using inhaled NDS.
			0	Current smoker
			Subtract 20 points (unless score is 0) for living with active indoor smoker in home	
	Sleep health	Self-reported average hours of sleep per night	Metric: Average hours of sleep per night	
			Scoring:	
			Points	Level
			100	7–<9
			90	9–<10
			70	6–<7
			40	5–<6 or ≥10
			20	4–<5
			0	<4
Health Factors	Body mass index	Body weight (kg) divided by height squared (m^2^)	Metric: Body mass index (kg/m^2^)	
			Scoring:	
			Points	Level
			100	<25
			70	25.0–29.9
			30	30.0–34.9
			15	35.0–39.9
			0	≥40.0
	Blood lipids	Plasma total and HDL-cholesterol with calculation of non-HDL-cholesterol	Metric: Non-HDL-cholesterol (mg/dL)	
			Scoring:	
			Points	Level
			100	<130
			60	130–159
			40	160–189
			20	190–219
			0	≥220
			If drug-treated level, subtract 20 points	
	Blood glucose	Fasting blood glucose or casual hemoglobin A1c	Metric: Fasting blood glucose (mg/dL) or Hemoglobin A1c (%)	
			Scoring:	
			Points	Level
			100	No history of diabetes and FBG <100 (or HbA1c <5.7)
			60	No history of diabetes and FBG 100–125 (or HbA1c 5.7–6.4)
			40	Diabetes with HbA1c <7.0
			30	Diabetes with HbA1c 7.0–7.9
			20	Diabetes with HbA1c 8.0–8.9
			10	Diabetes with HbA1c 9.0–9.9
			0	Diabetes with HbA1c ≥10.0
	Blood pressure	Appropriately measured systolic and diastolic blood pressure	Metric: Systolic and diastolic blood pressure (mm Hg)	
			Scoring:	
			Points	Level
			100	<120/<80 (Optimal)
			75	120–129/<80 (Elevated)
			50	130–139 or 80–89 (Stage I HTN)
			25	140–159 or 90–99
			0	≥160 or ≥100
			Subtract 20 points if treated level	

LE8, Life’s Essential 8; CVH, cardiovascular health; HDL, high-density lipoprotein; FBG, fasting blood glucose; HbA1c, hemoglobin.

### 2.4 Covariate Assessments

Drawing upon prior studies, a set of relevant covariates was identified for adjustment, including demographic characteristics (age, sex, race/ethnicity, education level, and marital status), socioeconomic status (PIR), and clinical or behavioral factors such as BMI, tobacco use, alcohol intake, and histories of stroke, myocardial infarction, and Cardiovascular Disease (CVD). Race and ethnicity were categorized into five groups: non-Hispanic White, non-Hispanic Black, Mexican American, other Hispanic individuals, and other racial or ethnic backgrounds. Educational level was stratified as less than high school, high school graduate, or education beyond high school. Marital status was classified as married or cohabiting, never married, and widowed, divorced, or separated. The PIR was divided into tertiles: ≤1.5, 1.5–3.5, and >3.5. BMI was grouped into four clinical categories: underweight (<18.5 kg/m^2^), normal weight (18.5–24.9 kg/m^2^), overweight (25.0–29.9 kg/m^2^), and obese (≥30 kg/m^2^). Smoking status was grouped into three types: former smoker, never smoker, and current smoker. Alcohol consumption was divided into five categories: former drinker, heavy drinker, light drinker, moderate drinker, and never drinker. Histories of stroke, myocardial infarction, and CVD were documented as binary variables (yes/no) [22, 23, 24].

### 2.5 Statistical Analysis

Because NHANES features an elaborate design, we weighted all analyses in accordance with its analytical procedures to make sure the sample accurately mirrors the entire USA. population. In the baseline analysis, continuous variables were summarized as means with standard errors, while categorical variables were presented as proportions. Group comparisons for continuous data were performed using the *t*-test, and for categorical variables using the χ^2^ test. To reduce selection bias and improve group comparability, a 1:2 propensity score matching (PSM) approach was applied, wherein each PD case was matched with two non-PD controls. This matching ratio is commonly utilized in observational studies, as it improves statistical efficiency while minimizing confounding [25, 26, 27]. Multivariable logistic regression analyses were performed to evaluate the association between LE8 and the prevalence of PD. Initially, LE8 was treated as a continuous variable. Subsequently, for categorical analysis, LE8 scores were stratified into three levels: poor CVH (0–49), intermediate (50–79), and optimal (80–100). Survey-weighted logistic regression models were employed, adjusting relevant covariates.

Three sequential logistic regression models were developed. Model 1 included no covariate adjustments. Model 2 controlled for demographic and socioeconomic factors, including age, sex, race/ethnicity, marital status, educational attainment, and income level. Model 3 extended the adjustment by additionally incorporating clinical history variables such as stroke, myocardial infarction, and CVD. To assess potential nonlinear associations between LE8 and PD, restricted cubic spline (RCS) functions were utilized for flexible curve fitting. Additionally, stratified subgroup analyses were carried out based on age, sex, race, educational background, income level, smoking and drinking behavior, and histories of stroke, heart attack, and CVD—both before and after PSM [28].

All statistical analyses were conducted in R (version 4.2.2; R Core Team, R Foundation for Statistical Computing, Vienna, Austria). Data preparation utilized the “nhanesR” package (https://rdrr.io/github/yikeshu0611/nhanesR), while the “survey” package was applied for weighted logistic regression. Statistical significance was determined based on a two-tailed *p*-value threshold of <0.05.

## 3. Results

### 3.1 Baseline Characteristics of NHANES Participants Between 2005 and 2018

Table 2 summarizes the baseline characteristics of NHANES participants from 2005 to 2018, stratified by PD status. After excluding individuals with incomplete key variables, 259 participants were identified as PD cases, accounting for 1.4% of the total sample. The average age of PD participants was 64.6 years, notably older than the non-PD group (59.6 years). A greater proportion of PD participants were female (55%) and non-Hispanic White (67%).

**Table 2.** **Characteristics of the study population from NHANES 2005–2018 before matching**.

Characteristic		Overall, n (%)	Non-PD, n (%)	PD, n (%)	*p* value
		N = 18,270 (100)	N = 18,011 (99)	N = 259 (1.4)	
Age (years)		59.7 (12.2)	59.6 (12.2)	64.6 (13.0)	<0.001
Sex					0.3
	Female	9441 (52)	9299 (52)	142 (55)	
	Male	8829 (48)	8712 (48)	117 (45)	
Race					<0.001
	Non-Hispanic white	8848 (48)	8674 (48)	174 (67)	
	Non-Hispanic black	3844 (21)	3810 (21)	34 (13)	
	Mexican American	2429 (13)	2403 (13)	26 (10)	
	Other Hispanic	1610 (8.8)	1591 (8.8)	19 (7.3)	
	Other race	1539 (8.4)	1533 (8.5)	6 (2.3)	
Poverty Ratio					0.003
	<1.3	5047 (28)	4958 (28)	89 (34)	
	1.3–3.5	7008 (38)	6900 (38)	108 (42)	
	>3.5	6215 (34)	6153 (34)	62 (24)	
Education					0.2
	Over High School	9546 (52)	9414 (52)	132 (51)	
	Below High School	4452 (24)	4378 (24)	74 (29)	
	High School	4272 (23)	4219 (23)	53 (20)	
Marital Status					0.2
	Married/living with partner	11,448 (63)	11,296 (63)	152 (59)	
	Widowed/divorced/separated	5384 (29)	5304 (29)	80 (31)	
	Never married	1438 (7.9)	1411 (7.8)	27 (10)	
Smoke					0.7
	Never	9321 (51)	9190 (51)	131 (50.6)	
	Former	5698 (31.2)	5621 (31.2)	77 (29.7)	
	Now	3251 (17.8)	3200 (17.8)	51 (19.7)	
Alcohol					<0.001
	Former	3841 (21)	3763 (20.9)	78 (30.1)	
	Heavy	2473 (13.5)	2452 (13.6)	21 (8.1)	
	Mild	6869 (37.7)	6769 (37.6)	100 (38.6)	
	Moderate	2543 (13.9)	2521 (14)	22 (8.5)	
	Never	2544 (13.9)	2506 (13.9)	38 (14.7)	
BMI					0.022
	Obese (30 or greater)	7498 (41)	7371 (40.9)	127 (49)	
	Overweight (25 to <30)	6320 (34.6)	6254 (34.7)	6 (25.5)	
	Normal (18.5 to <25)	4237 (23.2)	4174 (23.2)	63 (24.3)	
	Underweight (<18.5)	215 (1.2)	212 (1.2)	3 (1.2)	
Stroke					<0.001
	No	17,194 (94.1)	16,972 (94.2)	222 (85.7)	
	Yes	1076 (5.9)	1039 (5.8)	37 (14.3)	
Heart Attack					<0.001
	No	17,088 (93.5)	16,860 (93.6)	228 (88)	
	Yes	1182 (6.5)	1151 (6.4)	31 (12)	
CVD					<0.001
	No	15,325 (83.9)	15,153 (84.1)	172 (66.4)	
	Yes	2945 (16.1)	2858 (15.9)	87 (33.6)	
LE8		63.28 (14.27)	63.35 (14.26)	58.43 (14.75)	<0.001

BMI, Body Mass Index; CVD, Cardiovascular Disease.

In the PD group, 42% had a poverty-income ratio between 1.3 and 3.5, while 51% reported education beyond high school. Marital status indicated that 59% were either married or living with a partner. Additionally, 51% of individuals with PD had never smoked, and 38% reported light alcohol intake. Nearly half (49%) were categorized as obese. The prevalence of stroke, myocardial infarction, and CVD in the PD group was 14%, 12%, and 34%, respectively. Notably, the mean LE8 score was lower in PD participants (58.43) than in non-PD participants (63.35).

After PSM matching, as shown in Table 3, a total of 777 participants were included: 259 in the PD group and 518 in the non-PD group. The PD and non-PD groups showed similarity across age, sex, race, education level, marital status, poverty level, BMI, smoking status, alcohol consumption, stroke, heart attack, and CVD. For LE8 scores, the non-PD group had a mean of 62.39, which was higher than the PD group’s mean of 58.43.

**Table 3.** **Characteristics of the study population from NHANES 2005–2018 after matching**.

Characteristic		Overall, n (%)	Non-PD, n (%)	PD, n (%)	*p* value
		N = 777 (100)	N = 518 (67)	N = 259 (33)	
Age (years, meae (SD))		65.1 (12.5)	65.4 (12.3)	64.6 (13.0)	0.41
Sex					0.6
	Female	416 (54)	274 (53)	142 (55)	
	Male	361 (46)	244 (47)	117 (45)	
Race					0.7
	Non-Hispanic white	532 (68)	358 (69)	174 (67)	
	Non-Hispanic black	107 (14)	73 (14)	34 (13)	
	Mexican American	74 (9.5)	48 (9.3)	26 (10)	
	Other Hispanic	45 (5.8)	26 (5.0)	19 (7.3)	
	Other Race	19 (2.4)	13 (2.5)	6 (2.3)	
Poverty Ratio					0.6
	<1.3	251 (32)	162 (31)	89 (34)	
	1.3–3.5	325 (42)	217 (42)	108 (42)	
	>3.5	201 (26)	139 (27)	62 (24)	
Education					0.9
	Below High School	214 (28)	140 (27)	74 (29)	
	High School	158 (20)	105 (20)	53 (20)	
	Over High School	405 (52)	273 (53)	132 (51)	
Marital status					0.3
	Married/living with partner	482 (62)	330 (64)	152 (59)	
	Never married	68 (8.8)	41 (7.9)	27 (10)	
	Widowed/divorced/separated	227 (29)	147 (28)	80 (31)	
Smoke					0.0046
	Never	392 (50)	261 (50)	131 (51)	
	Former	239 (31)	162 (31)	77 (30)	
	Now	146 (19)	95 (18)	51 (20)	
Alcohol					0.2
	Former	195 (25.1)	117 (22.6)	78 (30.1)	
	Heavy	72 (9.3)	51 (9.8)	21 (8.1)	
	Mild	304 (39)	204 (39.4)	100 (38.6)	
	Moderate	75 (9.7)	53 (10.2)	22 (8.5)	
	Never	131 (16.9)	93 (18)	38 (14.7)	
BMI					0.13
	Obese (30 or greater)	332 (44)	210 (41)	122 (49)	
	Overweight (25 to <30)	233 (31)	169 (33)	64 (26)	
	Normal (18.5 to <25)	187 (25)	126 (25)	61 (24)	
	Underweight (<18.5)	8 (1.1)	5 (1.0)	3 (1.2)	
Stroke					>0.9
	No	667 (86)	445 (86)	222 (86)	
	Yes	110 (14)	73 (14)	37 (14)	
Heart Attack					>0.9
	No	684 (88)	456 (88)	228 (88)	
	Yes	93 (12)	62 (12)	31 (12)	
CVD					0.7
	No	508 (65)	336 (65)	172 (66)	
	Yes	269 (35)	182 (35)	87 (34)	
LE8 (meae (SD))		61.07 (14.43)	62.39 (14.10)	58.43 (14.75)	<0.001

### 3.2 Association Between LE8 and PD

Table 4 displays the associations between LE8 scores and PD prevalence across the three logistic regression models.

**Table 4.** **Multivariable logistics regression analysis of the association between LE8 and PD**.

Model	Characteristic		Unmatching		Matching	
			OR (95% CI)	*p*-value	OR (95% CI)	*p*-value
Model 1	LE8		0.98 (0.97, 0.98)	<0.0001	0.98 (0.96, 0.99)	0.002
	LE8 Category					
		Low (<50)	ref	ref	ref	ref
		Moderate (50–79)	0.53 (0.36, 0.78)	0.001	0.54 (0.33, 0.89)	0.02
		High (≥80)	0.34 (0.16, 0.69)	0.003	0.34 (0.15, 0.79)	0.01
Model 2	LE8		0.98 (0.96, 0.99)	<0.001	0.98 (0.96, 0.99)	0.01
	LE8 Category					
		Low (<50)	ref	ref	ref	ref
		Moderate (50–79)	0.58 (0.38, 0.87)	0.01	0.54 (0.31, 0.94)	0.03
		High (≥80)	0.39 (0.18, 0.83)	0.01	0.34 (0.14, 0.86)	0.02
Model 3	LE8		0.98 (0.97, 0.99)	0.002	0.97 (0.96, 0.99)	0.01
	LE8 Category					
		Low (<50)	ref	ref	ref	ref
		Moderate (50–79)	0.62 (0.40, 0.96)	0.03	0.52 (0.30, 0.90)	0.02
		High (≥80)	0.43 (0.20, 0.94)	0.01	0.32 (0.13, 0.81)	0.02

In Model 1, each one-point increment in LE8 was associated with a 2% decrease in PD risk prior to matching (OR = 0.98, *p* < 0.0001) and a 1% reduction after matching (OR = 0.98, *p* = 0.002). Compared with participants whose LE8 score was <50, those scoring 50–79 exhibited markedly lower odds of PD—47% before matching (OR = 0.53, *p* = 0.001) and 46% after matching (OR = 0.54, *p* = 0.02). The strongest inverse association was observed for scores ≥80, corresponding to a 66% risk reduction both before (OR = 0.34, *p* = 0.003) and after matching (OR = 0.34, *p* = 0.01).

Model 2 yielded comparable findings. Each one-point rise in LE8 was linked to a 2% reduction in PD risk both before (OR = 0.98, *p* < 0.001) and after matching (OR = 0.98, *p* = 0.01). Relative to individuals in the lowest LE8 category (<50), those with moderate scores (50–79) showed a 42% lower risk prior to matching (OR = 0.58, *p* = 0.01) and a 46% reduction following matching (OR = 0.54, *p* = 0.03). Participants with high LE8 scores (≥80) experienced a 61% decrease in PD risk before matching (OR = 0.39, *p* = 0.01) and a 66% reduction afterward (OR = 0.34, *p* = 0.02).

Model 3, which incorporated additional adjustments for clinical variables including stroke, myocardial infarction, and CVD, demonstrated similar trends. Each one-point increase in LE8 was associated with a 2% reduction in PD risk prior to matching (OR = 0.98, *p* = 0.002) and a 3% reduction following matching (OR = 0.97, *p* = 0.01). Compared to the low LE8 group (<50), participants with moderate scores (50–79) exhibited a 38% lower PD risk before matching (OR = 0.62, *p* = 0.03) and a 48% reduction after matching (OR = 0.52, *p* = 0.02). Individuals in the high LE8 category (≥80) showed the greatest benefit, with a 57% decrease in PD risk pre-matching (OR = 0.43, *p* = 0.01) and a 68% reduction post-matching (OR = 0.32, *p* = 0.02).

Additionally, domain-specific analyses were conducted to identify which individual LE8 components were most robustly linked to PD risk. Following adjustment for age, sex, and race, it was observed that diet quality (OR per 10-point increase: 0.91, *p* < 0.001) and sleep health (OR: 0.91, *p* < 0.001) acted as the strongest protective factors. Physical activity (OR: 0.95, *p* = 0.001) and BMI (OR: 0.95, *p* = 0.015) were also significantly linked to a lower PD risk. Other domains, including nicotine exposure and blood glucose, exhibited borderline significance (*p* = 0.050), whereas blood pressure and non-HDL cholesterol showed no significant association with PD risk. These findings are presented in Table 5.

**Table 5.** **Association between individual LE8 components and PD risk**.

LE8 Component	OR (95% CI)	*p*-value
Diet	0.91 (0.88–0.95)	<0.001
Sleep health	0.91 (0.87–0.95)	<0.001
Physical activity	0.95 (0.92–0.98)	0.001
Body-mass index (BMI)	0.95 (0.91–0.99)	0.015
Nicotine exposure	0.97 (0.94–1.00)	0.050
Blood glucose	0.96 (0.92–1.00)	0.050
Blood pressure	0.99 (0.95–1.03)	0.620
Non-HDL cholesterol	1.03 (0.98–1.07)	0.130

Adjusted for age, sex, and race/ethnicity. Odds ratios (ORs) represent the effect per 10-point increase in each LE8 component score.

### 3.3 Nonlinear Dose-Response Relationship Between LE8 and PD Prevalence

**Fig. 2.**
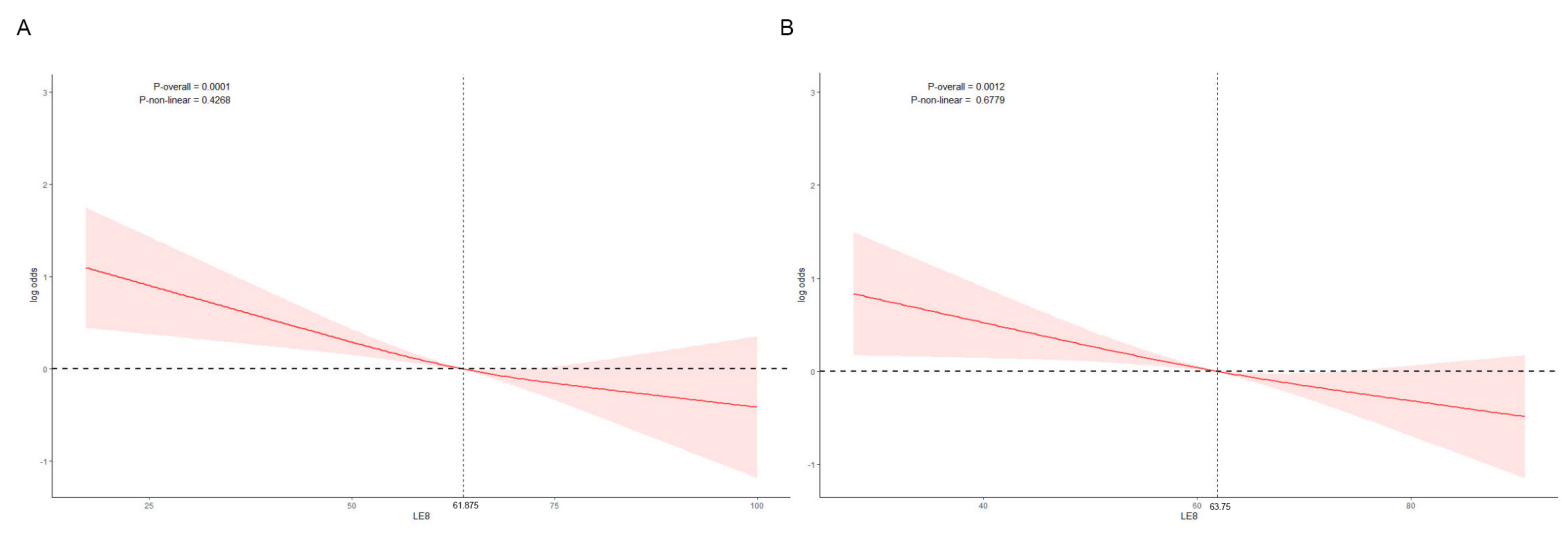
**Restricted cubic spline (RCS) analysis of the association between LE8 score and Parkinson’s disease (PD) risk**. (A) Before propensity score matching. (B) After propensity score matching. The red line represents the odds ratio (log-transformed) of PD associated with the LE8 score, and the shaded area indicates the 95% confidence interval.

Fig. 2 illustrates the non-linear association between LE8 scores and PD risk, modeled using RCS within a covariate-adjusted, weighted logistic regression framework. In Fig. 2A (before PSM), an inverse but non-linear relationship was observed: PD risk decreased with increasing LE8 scores, but this decline plateaued at approximately 61.88. In Fig. 2B (after matching), the non-linear pattern persisted. A significant reduction in PD risk was again noted with higher LE8 values, although the decreasing trend leveled off around a score of 63.75.

### 3.4 Subgroup Analysis Before and After Matching

**Fig. 3.**
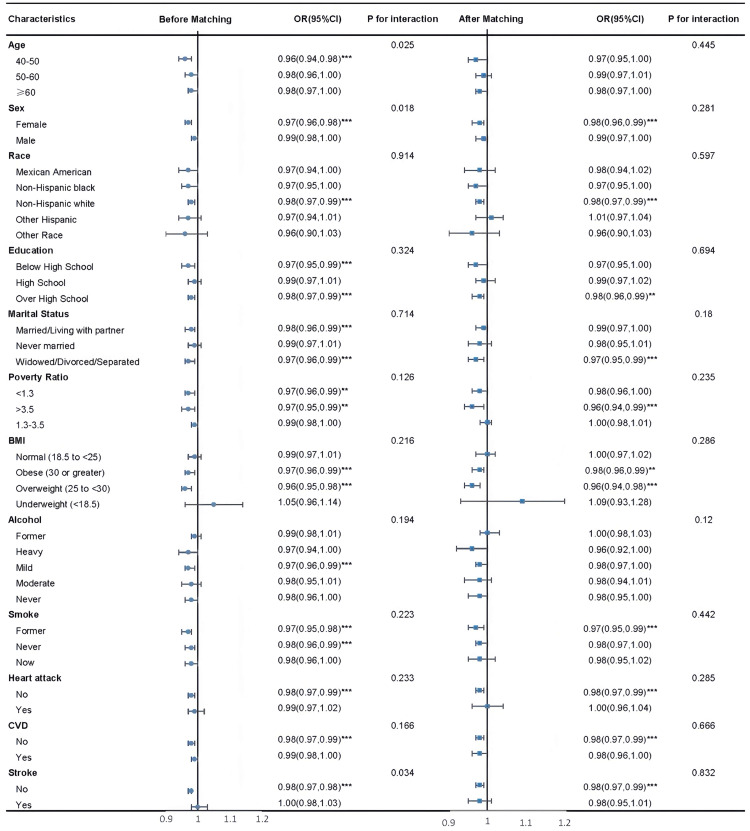
**Subgroup analysis before and after matching**. ***p < 0.001; **p < 0.01.

To evaluate potential effect modification, subgroup analyses were conducted both before and after PSM (Fig. 3). Stratification was based on age, sex, race/ethnicity, educational attainment, marital status, PIR, BMI, smoking and alcohol use, as well as histories of stroke, myocardial infarction, and CVD.

Prior to matching, significant inverse associations between LE8 scores and PD risk were identified in several subgroups, including individuals aged 40–50, females, those with either less than or more than high school education, and participants who were married/cohabiting or widowed/divorced/separated. Protective associations were also evident among those with PIR <1.3, overweight or obese BMI, light alcohol intake, former smokers, and individuals without a history of CVD or stroke.

After matching, these associations generally weakened, with odds ratios trending toward null (OR ≈ 1.00) in many subgroups. Nonetheless, the inverse relationship remained evident in certain strata, such as participants aged 40–50, females, individuals with at least a high school education, those married or formerly married, PIR <1.3, overweight individuals, moderate drinkers, former smokers, and those with a history of heart disease. Although statistical significance was reduced, higher LE8 scores continued to correspond with lower PD risk across these groups.

## 4. Discussion

### 4.1 Main Findings

This nationally representative analysis revealed a significant inverse relationship between LE8 scores and PD risk. Individuals with higher LE8 scores exhibited a consistently lower likelihood of PD, even after controlling for confounding factors through PSM. In particular, the tertile-based comparison demonstrated that participants in the highest CVH category (scores 80–100) had a markedly reduced risk relative to those in the lowest category (scores <50).

The overall association between LE8 and PD risk exhibited a nonlinear pattern: as LE8 scores increased, PD risk decreased, with this downward trend leveling off when LE8 scores reached 61.88 (pre-matching) and 63.75 (post-matching).

Within subgroup analyses, despite some attenuation in significance, an inverse correlation between LE8 and PD prevalence persisted.

### 4.2 Comparison With Other Studies and Potential Mechanisms

Among all PD cases, a small proportion of familial PD is associated with monogenic mutations [29, 30]. The majority of other PD cases are sporadic, resulting from gene-environment interactions [31]. Among various environmental factors, lifestyle has attracted significant scientific attention [32, 33, 34]. LE8 integrates diet, smoking, PA, BMI, blood pressure, total cholesterol, fasting blood glucose (FBG), and sleep, comprehensively considering lifestyle factors. Evidence exists indicating that specific dietary components can reduce PD risk [35, 36]. Studies have shown that individuals with low to moderate beer consumption exhibit a lower PD risk, whereas heavy drinkers show a higher risk [37]. Research indicates that BMI is negatively correlated with disease duration and severity [38]. Among males, a dose-dependent relationship exists between dietary cholesterol and lower PD risk [39]. Smoking is negatively correlated with PD [40], as are physical activity and energy intake [41]. These findings are consistent with prior evidence from mechanistic and pathological perspectives. The development of PD involves a multifactorial interplay of biological disturbances, including aberrant α-synuclein accumulation, mitochondrial impairment, disruptions in lysosomal or vesicular trafficking, synaptic transport abnormalities, and chronic neuroinflammation [42, 43]. In terms of diet, the onset of PD may be related to mitochondrial dysfunction. Vitamin E can improve mitochondrial and lysosomal function, thereby alleviating PD symptoms. Specifically, vitamin E acts as a potent antioxidant that reduces reactive oxygen species (ROS), protects mitochondrial membranes from oxidative damage, and supports lysosomal degradation of misfolded proteins such as α-synuclein, thereby mitigating neurodegeneration in PD [44]. Additionally, a ketogenic diet has shown significant efficacy in treating PD. The neuroprotective effects induced by ketone bodies result from increased mitochondrial respiration through enhanced adenosine triphosphate (ATP) production [45]. Polyunsaturated fatty acids, such as arachidonic acid, docosahexaenoic acid (DHA), and eicosapentaenoic acid (EPA), can enhance neuronal cell membrane excitability while inhibiting free radical production and reducing inflammation [46].

Regarding physical activity, the precise mechanisms by which it alters the onset and progression of PD are not fully understood. However, exercise-induced improvements in insulin signaling, inflammation, mitochondrial dysfunction, and endoplasmic reticulum stress may promote the survival of dopaminergic neurons by altering the expression of α-synuclein, inflammasomes, and neurotrophic factors [47, 48]. In terms of sleep, sleep abnormalities are recognized as common non-motor features of PD [49]. Beyond early symptoms of neurodegeneration, sleep disturbances may play a critical role in PD pathogenesis, given that slow-wave sleep and rapid eye movement sleep are severely disrupted [50]. Sleep plays a crucial role in waste clearance, with amyloid proteins and other harmful solutes being cleared from the brain at higher rates [51]. Sleep deprivation and fragmentation-induced lymphatic system dysfunction are associated with increased α-synuclein deposition in the substantia nigra, exacerbating the neuropathological progression of PD [52]. Smoking and alcohol consumption may saturate a significant proportion of nicotinic receptors in the brain, thereby exerting neuroprotective effects [53]. Moreover, recent studies suggest that excessive iron accumulation in brain regions such as the substantia nigra may contribute to both neurodegeneration and cognitive impairment in PD through oxidative stress and neuroinflammatory pathways [54, 55]. Incorporating this mechanism may provide further insight into the progression of non-motor symptoms and highlight potential new directions for prevention and therapy.

Prior research has documented links between LE8 and stroke, mood disorders (e.g., depression), and other chronic conditions [56, 57, 58]. Additionally, some studies have reported associations between lifestyle factors and PD [59]. However, the present study represents the first to select PD patients from the nationally representative NHANES sample, comprehensively account for lifestyle factors via LE8 scores, and systematically assesses the relationship between LE8 and PD. Results from this study indicate a significant inverse correlation between LE8 and PD.

### 4.3 Strengths and Limitations

This study presents several strengths. Most notably, it leveraged data from a nationally representative U.S. sample, with NHANES providing high-quality, standardized data collection protocols that enhance the external validity of the results. The relationship between LE8 and PD was rigorously assessed using PSM, and non-linear trends were further examined through RCS modeling, allowing for a nuanced evaluation of the dose-response pattern.

Nevertheless, certain limitations warrant consideration. Due to the cross-sectional nature of the NHANES dataset, causal relationships between LE8 scores and PD risk cannot be established. Reverse causality remains a potential concern, as prodromal symptoms—such as impaired sleep, reduced physical activity, or metabolic alterations—may precede PD diagnosis and adversely influence LE8 scores. For instance, early fatigue or sleep disruption may directly reduce scores in relevant LE8 domains. Thus, it remains unclear whether suboptimal CVH contributes to PD onset or if early disease manifestations affect lifestyle-related metrics. Longitudinal studies are essential to clarify this temporal relationship. Second, the reliance on self-reported data for both PD status and several LE8 components introduces the possibility of recall bias and misclassification. Third, PD identification was based on the use of anti-Parkinsonian medications, as NHANES lacks clinical diagnoses or ICD coding. While this method is consistent with prior NHANES-based research, it may misclassify individuals receiving such medications for alternative conditions (e.g., restless legs syndrome) or those not yet undergoing pharmacologic treatment. Lastly, the relatively small number of PD cases limits statistical power, and future research with larger, clinically verified cohorts is needed to confirm these findings.

## 5. Conclusion

Findings from this cross-sectional analysis suggest that LE8, a composite measure of lifestyle-related factors, is inversely associated with the prevalence of PD. Individuals with higher LE8 scores were less likely to report PD. However, due to the observational and cross-sectional nature of the data, these results reflect correlation rather than causation, and no definitive conclusions regarding temporal or causal relationships can be drawn.

## Availability of Data and Materials

Data is provided within the manuscript. The database used for this study can be found in online repositories (https://www.cdc.gov/nchs/nhanes/index.htm).
